# Parapharyngeal meningioma extending through foramen ovale: a case report

**DOI:** 10.3389/fonc.2023.1236066

**Published:** 2023-07-24

**Authors:** Jin Mao, Kai Wei, Siyu Yang, Ling Hu, Chao Wang

**Affiliations:** ^1^ Department of Radiology, The Second Affiliated Hospital, Zhejiang University School of Medicine, Hangzhou, China; ^2^ Department of Ultrasound, Hangzhou Women’s Hospital, Hangzhou, Zhejiang, China

**Keywords:** extracranial meningioma, parapharyngeal space, foramen ovale, magnetic resonance imaging, preoperative diagnosis

## Abstract

**Background:**

Meningioma is a common non-glial tumor of the brain. Extracranial meningiomas in the parapharyngeal space are especially rare. Herein we report a case of extracranial meningioma in the parapharyngeal space and give a comprehensive description of its complete clinical course and radiological findings, which may provide helpful information in the diagnosis and treatment of extracranial meningiomas in the parapharyngeal space.

**Case Presentation:**

A 61-year-old man presented a slowly increased mass under the left ear without pain and numbness over one year. Ultrasound examination detected a hypoechoic uneven mass behind the left parotid gland with a clear boundary, and color Doppler flow imaging revealed blood flow signals within the mass. Unenhanced computed tomography (CT) of the craniofacial region revealed a homogenous soft tissue mass in the parapharyngeal space without calcification. Magnetic resonance imaging (MRI) showed that a homogenous soft tissue mass was hyperintense on T2-weighted image, hypointense on T1-weighted image, and obviously enhanced after contrast enhancement in the parapharyngeal space. Coronal MRI showed that the lesion originated from basicranial dura extending into parapharyngeal space through the left foramen ovale at the skull base. Finally, histopathological and immunohistochemical analyses confirmed the final diagnosis of extracranial meningiomas in the parapharyngeal space.

**Conclusion:**

Extracranial meningiomas of the parapharyngeal space are rare and often pose a diagnostic challenge. Preoperative imaging examinations, especially CT and MRI, can aid in the accurate preoperative diagnosis, especially when intracranial extensions and dural tail signs are observed.

## Introduction

Meningioma is a common non-glial tumor of the brain, accounting for 15% of all intracranial tumors (1). Meningioma originates from pia-arachnoid cells and is typically attached to the dura. Only 1-2% of the meningiomas are extracranial (2), such as the locations of the orbit and paranasal sinuses (3). Extracranial meningiomas in the parapharyngeal space are especially rare, with only a few published reports (4). Meningiomas in the parapharyngeal space may originate from the pluripotent mesenchymal cells, the meningocytic cell-rests of the cranial nerve sheaths. In addition, meningiomas in the parapharyngeal space can occur secondary to intracranial meningiomas that extend directly through the skull base foramina (5). Due to the rare nature and the lack of specific symptoms, the accurate preoperative diagnosis for parapharyngeal meningiomas is challenging. Herein, we present a rare case of parapharyngeal meningioma originating from basicranial dura extending into parapharyngeal space through the foramen ovale of the skull base. In addition, we reviewed the clinicopathological features, imaging features, and therapeutic strategies to provide helpful information in the diagnosis and treatment of extracranial meningiomas in the parapharyngeal space.

## Case presentation

**Figure 1:**
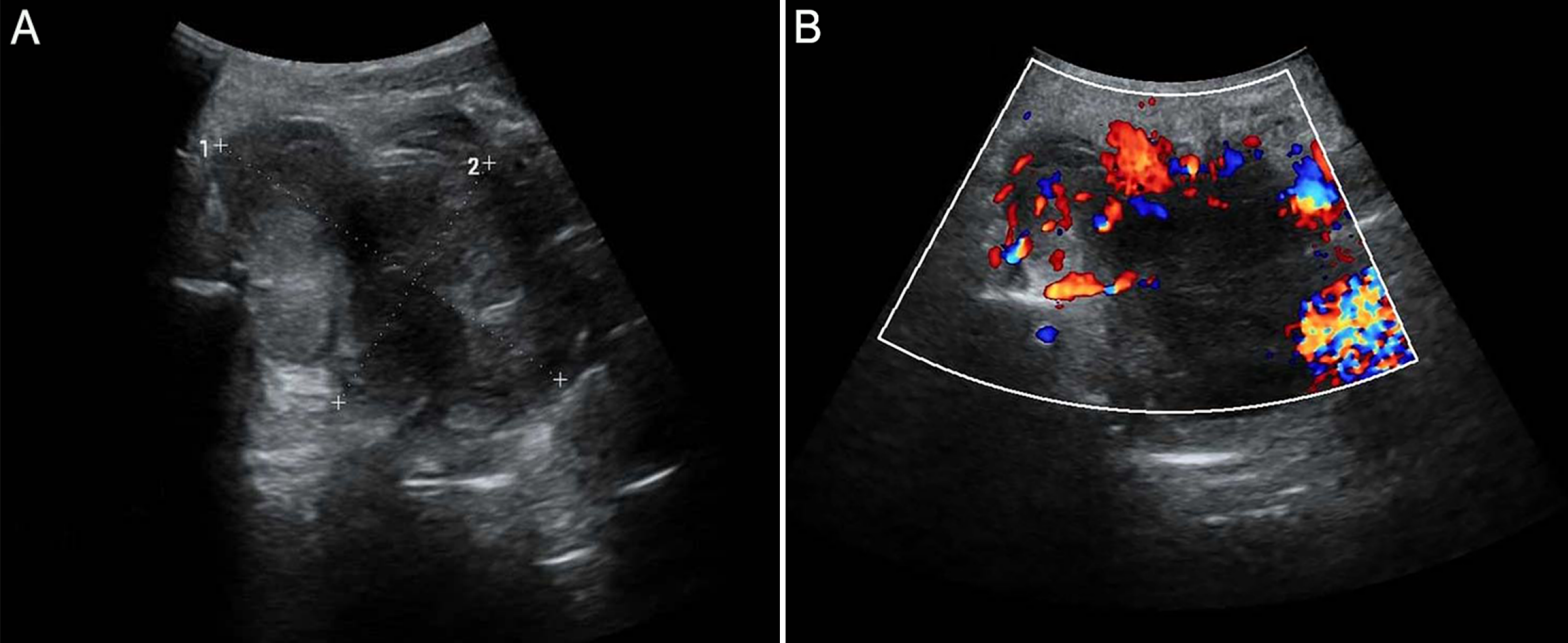
Ultrasound examination detects a hypoechoic uneven mass behind the left parotid gland with a clear boundary **(A)**, and color Doppler flow imaging (CDFI) reveals blood flow signals **(B)**.

**Figure 2:**
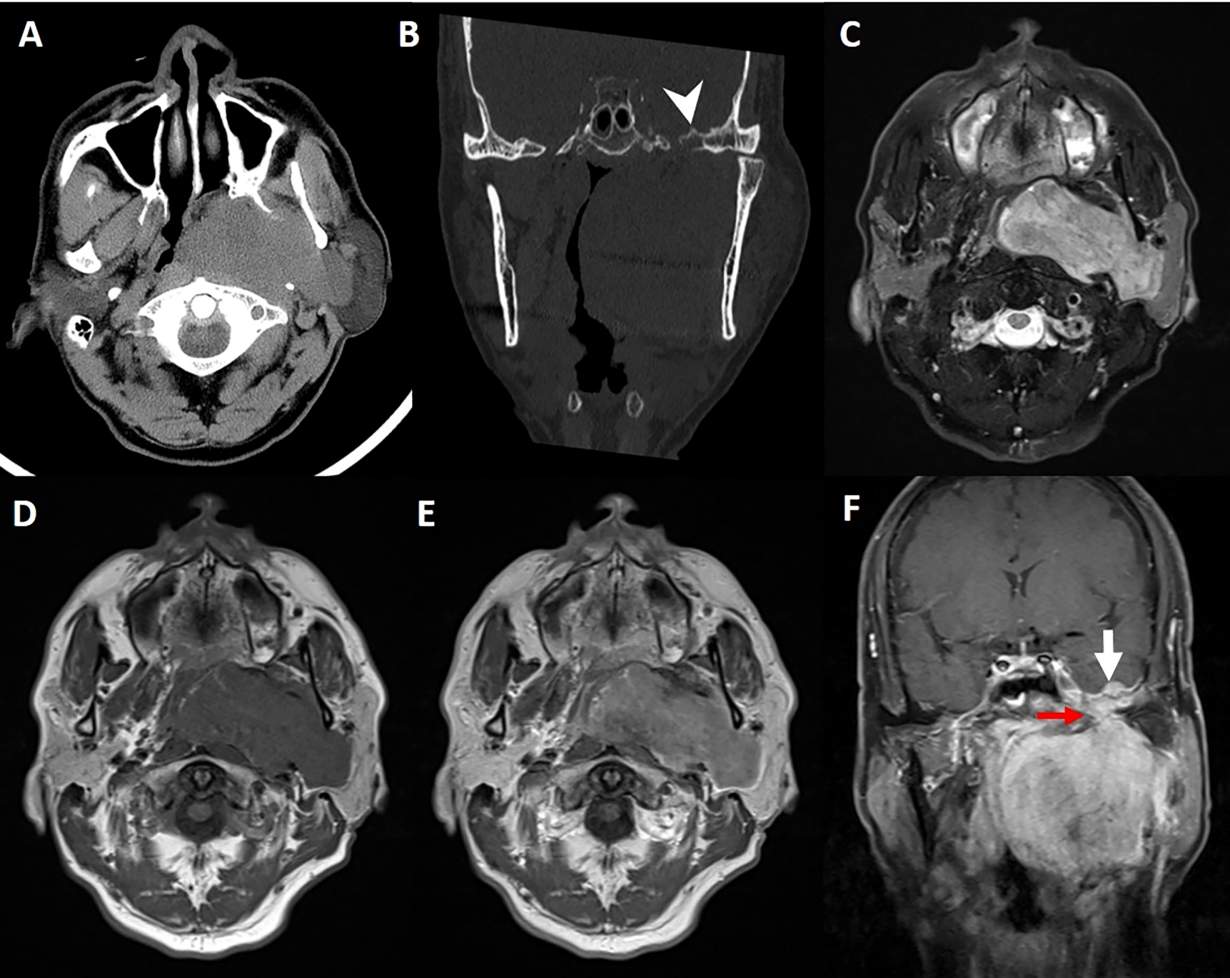
Axial **(A)** and coronal **(B)** CT images reveal locally rough, absorbed, and thinning changes on the left temporal bone (arrowhead). The lesion appears hyperintense with focal hypointense on T2-weighted image **(C)** with a sharply defined edge, homogenous isointense on unenhanced T1-weighted image **(D)**, and obviously homogenous enhancement on contrast-enhanced T1-weighted images **(E, F)**. Coronal gadolinium-enhanced T1-weighted image **(F)** demonstrates thickened basicranial dura with a nodule (thick white arrow) on the left middle cranial fossa connecting with a mass in the left parapharyngeal space through the foramen ovale (thin red arrow).

**Figure 3:**
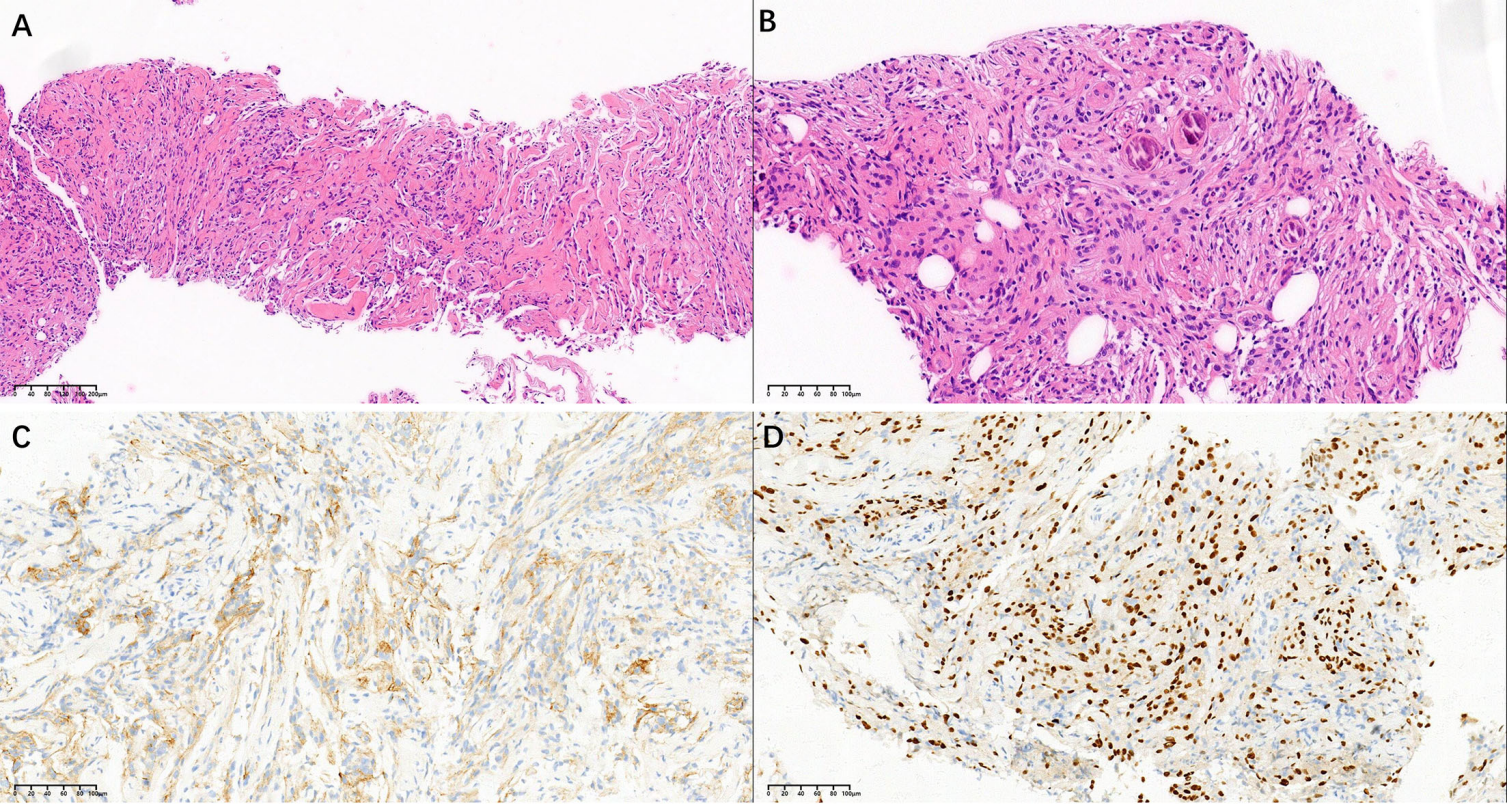
Histological staining images **(A, B)** show a spindle cell tumor with scattered psammoma bodies (HE, ×100 and ×200). Immunohistochemical staining images show that the tumor cells are positive for EMA **(C)** and PR **(D)** (×200).

**Figure 4:**
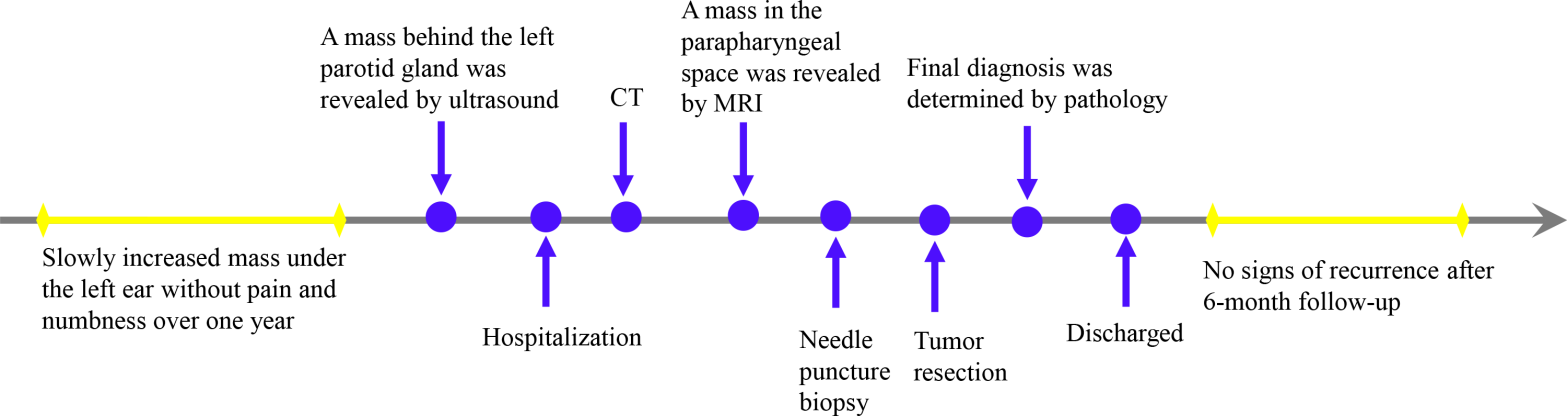
The timeline of diagnosis and treatment.

This study followed the tenets of the Declaration of Helsinki and was performed according to the guidelines of the Second Affiliated Hospital of Zhejiang University School of Medicine. Written informed consent was obtained from the patient. A 61-year-old man presented a slowly increased mass under the left ear without pain and numbness over one year. In addition, the patient bit the cheek with his left posterior teeth when he consciously ate over four months. The distension of the left lateral pharyngeal wall was visible in the mouth. Ultrasound examination detected a hypoechoic uneven mass (8.2*5.6cm in size) behind the left parotid gland with a clear boundary (**Figure 1A**), and color Doppler flow imaging (CDFI) revealed blood flow signals (**Figure 1B**) within the mass. Then, the initial diagnosis of pleomorphic adenoma of the parotid gland was made. Unenhanced computed tomography (CT) images of the craniofacial region revealed a homogenous soft tissue mass in the parapharyngeal space without calcification (**Figure 2A**). The left temporal bone revealed locally rough, absorbed, and thinning changes (**Figure 2B**). Magnetic resonance imaging (MRI) revealed that a homogenous soft tissue mass was hyperintense on the T2-weighted image (**Figure 2C**), hypointense on the T1-weighted image (**Figure 2D**), and obviously enhanced after contrast enhancement (**Figure 2E**) in the parapharyngeal space. The tumor size was 8.2cm (left and right) * 3.3cm (front and back) * 6.9cm (up and down). The surrounding structures were compressed by the mass. Coronal MRI image showed that the lesion originated from basicranial dura extending into parapharyngeal space through the left foramen ovale at the skull base (**Figure 2F**). A dural tail sign can be seen on the intracranial component, as well as a sharply defined edge on the extracranial component. Based on the CT and MRI imaging findings, neurogenic tumor and pleomorphic adenoma received diagnostic considerations for this lesion. The patient underwent an ultrasound-guided coarse needle puncture biopsy of the left parotid mass. Histopathology revealed a spindle cell tumor with scattered psammoma bodies (**Figures 3A, B**), suggesting a meningioma. Then, the patient underwent left basicranial tumor resection with partial parotidectomy. The postoperative pathology further confirmed that this was an extracranial meningioma in the parapharyngeal space. Immunohistochemical results showed CD117 +/-, CD34 +, Beta-catenin ++, smooth-muscle actin (SMA)-, epithelial membrane antigen (EMA)+ (**Figure 3C**), Ki-67 2%, P53 +/-, S-100 +, DOG-1 -, Desmin -, CK(AE1/AE3) -, glial fibrillary acidic protein (GFAP)-, progesterone receptor (PR)++ (**Figure 3D**). The patient recovered well and was discharged in a week in satisfactory condition after the operation. The patient was followed up by telephone half a year after the operation and reported no significant recurrence. The timeline of diagnosis and treatment is shown in **Figure 4**.

## Discussion

Extracranial meningiomas are very rare, comprising only 2% of all meningiomas. Due to the lack of prevalence and specific symptoms, extracranial meningiomas are often misdiagnosed (6). The most frequently reported extracranial sites of meningiomas are the head and neck, such as the sinonasal tract, ear/temporal bones, and orbit (7). Meningiomas rarely extend out of their intracranial confines through the skull foramen to present as a neck mass. Extension and presentation into parapharyngeal space are exceedingly rare (8). This study presents a rare parapharyngeal meningioma originating from basicranial dura extending into parapharyngeal space through the foramen ovale of the skull base.

The parapharyngeal space is enclosed superiorly by the skull base, posteriorly by the vertebral column, anteriorly by the mandible and parotid glands, and inferiorly by the submandibular glands. Parapharyngeal meningiomas present without specific symptoms. The mass often goes undetected for a long time because local symptoms do not exist until the mass reaches a significant size. This was the case with our patient who had a slowly increased mass under the left ear for more than one year without pain or numbness. When symptoms do occur, they can vary greatly depending on the site involved. The tumor may present as a mass in the neck, a bulge in the mouth, or a mass around the ear (9). Possible symptoms include neurological dysfunction, cranial nerve deficits, sinusitis, and proptosis (9).

CT and MRI examinations are essential diagnostic tools for parapharyngeal lesions and are very useful in preoperative surgical planning. The imaging diagnosis of parapharyngeal meningiomas is challenging because of the similarities among lymph node tumors, paragangliomas, and neurogenic tumors of the cranial nerves (10). Nevertheless, intracranial extensions and dural tail signs are conducive to identifying extracranial meningiomas, such as the case with our patient (**Figure 2F**).

Although most extracranial meningiomas in the parapharyngeal space are benign, surgical excision is the primary treatment. Pathological examination is essential in confirming a definite diagnosis. Histologically, extracranial meningiomas are similar to other classic meningiomas. Immunohistochemical pathology is a critical tool for confirming the diagnosis of an extracranial meningioma (11). In the case of our patient, the positive expressions of EMA, CD34, S100, and PR supported meningioma.

In summary, extracranial meningiomas in the parapharyngeal space are rare and often pose a diagnostic challenge. Preoperative imaging examinations, especially CT and MRI, can evaluate the tumor’s extent. These imaging modalities can aid in the accurate preoperative diagnosis, especially when intracranial extensions and dural tail signs are observed. Although the parapharyngeal region is a rare location, parapharyngeal meningioma should receive diagnostic consideration for the lesion that is a mass originating from the basicranial dura extending through the foramen at the skull base and showing a dural tail sign.

## Data availability statement

The original contributions presented in the study are included in the article/supplementary material. Further inquiries can be directed to the corresponding authors.

## Ethics statement

The studies involving human participants were conducted according to the guidelines of the Declaration of Helsinki and were reviewed and approved by the Medical Research Ethics Review Committee of The Second Affiliated Hospital, Zhejiang University School of Medicine. The patients/participants provided their written informed consent to participate in this study. Written informed consent has been obtained from the patient to publish this paper.

## Author contributions

Conceptualization, CW and LH. Methodology, JM and KW. Software, JM and KW. Investigation, JM and KW. Resources, SY and CW. Writing—original draft preparation, SY and CW. Writing—review and editing, LH and CW. Visualization, CW. Supervision, CW. Project administration, CW. All authors contributed to the article and approved the submitted version.

## References

[1] Louis DN Perry A Wesseling P Brat DJ Cree IA Figarella-Branger D. The 2021 WHO classification of tumors of the central nervous system: a summary. Neuro Oncol (2021) 23(8):1231–51. doi: 10.1093/neuonc/noab106 PMC8328013 34185076

[2] Gupta AP Parate RC. Fine-needle aspiration cytology of jugular foramen meningioma presenting as parapharyngeal mass. Indian J Pathol Microbiol (2011) 54(2):398–9. doi: 10.4103/0377-4929.81639 21623104

[3] Hallgrimsson J Björnsson A Gudmundsson G. Meningiona of the neck. Case report. J Neurosurg (1970) 32(6):695–9. doi: 10.3171/jns.1970.32.6.0695 5442596

[4] Albsoul N Rawashdeh B Albsoul A Abdullah M Golestani S Rawshdeh A. A rare case of extracranial meningioma in parapharyngeal space presented as a neck mass. Int J Surg Case Rep (2015) 11:40–3. doi: 10.1016/j.ijscr.2015.04.012 PMC4446679 25912007

[5] Uchibori M Odake G Ueda S Yasuda N Hisa I. Parapharyngeal meningioma extending from the intracranial space. Neuroradiology (1990) 32(1):53–5. doi: 10.1007/bf00593943 2333135

[6] Nager GT Heroy J Hoeplinger M. Meningiomas invading the temporal bone with extension to the neck. Am J Otolaryngol (1983) 4(5):297–324. doi: 10.1016/s0196-0709(83)80018-0 6416092

[7] Zakhari N Torres C Castillo M Nguyen TB. Uncommon cranial meningioma: key imaging features on conventional and advanced imaging. Clin Neuroradiol (2017) 27(2):135–44. doi: 10.1007/s00062-017-0583-y 28466126

[8] Taori K Kundaragi NG Disawal A Jathar C Gaur PP Rathod J. Imaging features of extra cranial parapharyngeal space meningioma: case report. Iran J Radiol (2011) 8(3):176–81. doi: 10.5812/kmp.iranjradiol.17351065.3132 PMC3522327 23329938

[9] Rushing EJ Bouffard JP McCall S Olsen C Mena H Sandberg GD. Primary extracranial meningiomas: an analysis of 146 cases. Head Neck Pathol (2009) 3(2):116–30. doi: 10.1007/s12105-009-0118-1 PMC2715454 19644540

[10] Shetty C Avinash KR Auluck A Mupparapu M. Extracranial meningioma of the parapharyngeal space: report of a case and review of the literature. Dentomaxillofac Radiol (2007) 36(2):117–20. doi: 10.1259/dmfr/56368887 17403892

[11] Swain RE Jr. Kingdom TT DelGaudio JM Muller S Grist WJ. Meningiomas of the paranasal sinuses. Am J Rhinol (2001) 15(1):27–30. doi: 10.2500/105065801781329419 11258651

